# Pilot study: predicting the interplay between FOXO1 and its downstream long non-coding RNAs in HCC

**DOI:** 10.3389/fonc.2026.1692980

**Published:** 2026-02-16

**Authors:** Rowan Abdelbary, Sherine K. Saber, Fabian Rose, Kai Breuhahn, Shereen A. El Sobky, Injie Omar Fawzy, Ahmed Moustafa, Nada El-Ekiaby, Ahmed Ihab Abdelaziz

**Affiliations:** 1 Biotechnology Graduate Program, American University in Cairo, Cairo, Egypt; 2 School of Medicine, Newgiza University (NGU), Giza, Egypt; 3 Institute of Pathology, University Hospital Heidelberg, Heidelberg, Germany; 4 Department of Biology, American University in Cairo, Cairo, Egypt

**Keywords:** ChIP-seq, forkhead box O-1 (FOXO1), hepatocellular carcinoma, long non-coding RNA, RNA sequencing

## Abstract

**Introduction:**

*Forkhead Box O-1* (*FOXO1*) is one of the key regulatory transcription factors capable of regulating many critical cellular functions, such as promoting apoptosis and inhibiting cell cycle progression thereby acting as a tumor-suppressor. In hepatocellular carcinoma (HCC), FOXO1 has been shown to be downregulated in liver tissues, and the lower expression has been correlated with poor prognosis. This highlights the necessity of understanding the regulatory functions of FOXO1 in more depth. Various studies have focused on the role of non-coding RNAs (ncRNAs), mainly microRNAs, as upstream regulators of FOXO1 in HCC and how their dysregulation affects carcinogenesis. However, the role of FOXO1 as an upstream regulator of ncRNAs, specifically long non-coding RNAs (lncRNAs), remains an unexplored area of research that needs to be addressed. In this study, we aimed to dissect this interplay and to identify potential FOXO1-regulated lncRNAs which could possibly play a role in the pathogenesis of HCC.

**Methods:**

This was achieved through the analysis of publicly available ChIP-Seq data provided by ENCODE database, along with performing RNA sequencing after the knockdown of FOXO1 in Huh-7 cells.

**Results:**

ChIP-Seq data analyses revealed a list of 982 promising lncRNAs that are possibly regulated by FOXO1. Analysis of the RNA sequencing data revealed 131 lncRNAs that were differentially expressed after FOXO1 knockdown. The intersection between ChIP-Seq data and RNA sequencing data showed an overall of 12 lncRNAs that were differentially expressed upon FOXO1 knockdown and also have a FOXO1 binding site in their promoter region, namely *ZFAS1, LINC00862, SNHG32, LINC01962, SNHG12, 1QCH-AS1, LINC00324, DCXR-DT*, *GLUD1P2, FAB5P3, JPX, and SMPD4BP*.

**Discussion:**

In conclusion, 12 lncRNAs were identified as potential downstream targets of FOXO1, suggesting that those lncRNAs could mediate FOXO1 functions in HCC.

## 1 Introduction

*Forkhead Box O-1* (*FOXO1*) is a transcription factor belonging to the FOXO family, known to regulate various biological functions, such as cell survival, differentiation, proliferation, glucose metabolism, longevity, and protection from oxidative stress (1, 2). The dysregulation of any of the FOXO1-regulated cellular processes threatens the integrity of cellular functions and is associated with the development of various disorders and diseases, such as metabolic disorders and cancers (1).

FOXO1 possesses a critical role in the regulation of liver functions. As a transcription factor, FOXO1 orchestrates the expression of genes which control many metabolic cascades, thereby regulating energy metabolism within the liver; these include genes involved in the processes of gluconeogenesis, glycogenolysis, and the metabolism of fatty acids (3–5). Furthermore, FOXO1 acts as a sensor for nutrient deficiency and starvation. For example, in fasting conditions FOXO1 suppression by insulin signaling was found to decrease glucose production through the downregulation of gluconeogenic genes (6). The dysregulation of FOXO1 in cases of diabetes and insulin resistance was shown to increase glucose production by the liver, resulting in hyperglycemia (6).

In addition to its metabolic role, FOXO1 plays a role in organ development and tumorigenesis, where it acts mainly as a tumor-suppressor by controlling cell proliferation, cell cycle progression, and programmed cell death via variable mechanisms such as oxidative stress protection and autophagy (7, 8). It has also been shown that FOXO1 plays a role in apoptosis, regulated by Akt signaling (9–11). Moreover, it controls cell cycle progression, as its activation promotes cyclin-dependent kinase inhibitor p27KIP1 expression, which results in cell cycle arrest (1, 12). Hence, the dysregulation of FOXO1 disrupts those processes, thereby inducing carcinogenesis.

In hepatocellular carcinoma (HCC), FOXO1 was found to be dysregulated by several mechanisms. Chand et al. have suggested that the aggressive progression of HCC is caused by the overexpression of FoxM1, which binds and inactivates retinoblastoma protein leading to the repression of FOXO1 (13). This enhances cell proliferation and inhibits cell senescence in HCC (13). The inactivation of FOXO1 is also mediated by the aberrant activation of PI3K/AKT signaling pathway in HCC, which leads to phosphorylation and subsequent inhibition of FOXO1, thereby boosting the proliferation of tumors, as it switches off cell death via apoptosis (7, 9, 14, 15). FOXO1 was also shown to suppress invasion of HCC cells and prevent lung metastasis in nude mice by reversing epithelial-mesenchymal transition (EMT) (16). Another study has shown that upon the overexpression of aquaporin 9, FOXO1 expression levels were increased in HCC cells, causing cell cycle arrest at the G_1_ phase with an increase in apoptosis and a decrease in proliferation (17). In addition, HCC patients with higher levels of FOXO1 were found to have better prognosis (18).

Collectively, these data draw attention to the critical role of FOXO1 in HCC. Therefore, it is crucial to grasp how FOXO1 levels are finetuned endogenously, prompting the focus towards the regulation of FOXO1 by non-coding RNAs (ncRNAs). Regulatory ncRNAs are key contributors in the modulation of gene expression, and include, among others, the microRNAs (miRNAs) and long non-coding RNAs (lncRNAs). Both miRNAs and lncRNAs are implicated in the pathogenesis of numerous diseases through their regulation of gene expression. miRNAs are small RNA molecules of an approximate size of 22 nucleotides, which associate with the 3’untranslated region (3’UTR) of their target transcripts, typically resulting in translational repression or transcript degradation (19). lncRNAs are more than 200 nucleotides long and include both linear and circular RNAs (circRNA) (20). lncRNAs can directly interact with chromatin, suppressing target gene expression (21). In addition to their direct interaction with chromatin, lncRNAs have also been shown to indirectly regulate gene expression through sponging miRNAs, thereby inhibiting their association with downstream target genes (22).

FOXO1 was shown to be regulated by several miRNAs, as well as a few lncRNAs, contributing to HCC pathogenesis. For instance, Zeng et al. have shown that miR-135a acts as an oncomiR, promoting the invasion and migration of HCC cells through the downregulation of FOXO1 (23). In addition, miR-196a inhibition caused an increase in FOXO1 expression, which resulted in the inhibition of cell proliferation and invasion through the activation of apoptosis (24). Moreover, miR-3174 and miR-1269 directly suppress FOXO1 leading to the enhancement of cell proliferation and the suppression of apoptosis (8, 11, 25). In addition, Lin et al. have found that miR-5188 suppressed the expression levels of FOXO1, leading to retention of β-catenin in the cytoplasm, and subsequent activation of the Wnt pathway, thus promoting tumorigenesis and EMT (26). Interestingly, the above-mentioned miRNAs were all reported to be upregulated in HCC patients (11, 26–28).

Besides miRNAs, a scarce number of studied lncRNAs were also shown to regulate FOXO1 indirectly through competitively sponging downstream miRNAs in HCC. For instance, tumor progression and glucose metabolism were suppressed due to the sponging of miR-183-5p by circRPN2 which led to the overexpression of FOXO1 (29). In addition, lncRNA *MEG3* was found to sponge miR-5195-3p leading to the induction of FOXO1 expression, subsequently resulting in the inhibition of HCC progression (30). It is noteworthy that while the regulation of FOXO1 by ncRNAs has received much attention, the regulation of ncRNAs by FOXO1 and their role in mediating FOXO1 functions remains an interesting niche that needs to be addressed. Hence, this study focused on ChIP-Seq analysis, along with RNA sequencing to identify lncRNAs that are potentially regulated by FOXO1 in HCC.

## 2 Materials and methods

### 2.1 FOXO1 Differential expression and survival analysis

The differential expression of FOXO1 in liver tissues of 374 HCC patients (cancer) and 50 controls (normal) was assessed using the PanCancer differential expression platform provided by Encyclopedia of RNA Interactomes (ENCORI) database, which relied on their analysis of RNA-Seq data available from The Cancer Genome Atlas (TCGA) database (31). The RNA-Seq data was presented as the gene expression values scaled with log2 (Fragments Per Kilobase of transcript per Million mapped reads (FPKM) + 0.01), while the miRNA expression values were scaled with log2 (Reads per million mapped reads (RPM) + 0.01) (31). Gene survival analysis was performed through ENCORI Gene survival analysis platform to determine the survival rate of patients with high vs. low FOXO1 expression (31).

### 2.2 ChIP-Seq data analysis

FOXO1 ChIP-Seq data acquired from liver cancer cells (HepG2) was retrieved from GEO (GSE170347) (32). The analysis was performed using the BED files, through which annotation of peaks was performed for FOXO1 utilizing the Bioconductor package ChIPseeker (33). Peak ranking was calculated by dividing the peak height over the peak width, and rankings falling below the first quartile were disregarded from subsequent analyses and considered background noise. Shortlisting criteria were set to include lncRNAs that have a FOXO1 binding site at their promoter region within the range of ±2000 nucleotides distance from the transcription start site (TSS).

### 2.3 FOXO1 knockdown in Huh-7 cells

Huh-7 cells were seeded at a density of 100,000 cells per well in a 6 well-plate and transfected with either custom-designed siRNAs targeting FOXO1 (siFOXO1) (GAG AUA AGC AAU CCC GAA A dT dT, CTM-59719, Dharmacon), or Allstars negative control oligonucleotides (NC) (1027292 Qiagen, Germany) at a concentration of 40nM the following day and incubated for 24 hours.

### 2.4 RNA extraction and quality check

After 24 hours of transfection with siRNAs, RNA extraction was performed using the miRNeasy column extraction method, according to the manufacturer’s instructions (217084, Qiagen, Germany). RNA quality and concentration were assessed through RNA integrity polyacrylamide gels and Qubit™ RNA HS Assay Kit (Qubit 3.0), respectively. Ribosomal RNA depletion was performed using RiboMinus™ Eukaryote Kit v2 (A15020, ThermoScientific, USA).

### 2.5 Next generation sequencing and shortlisting of FOXO1 downstream targets

Whole transcriptome library preparation was performed using the Ion Total RNA-Seq Kit v2 (4475936, ThermoScientific, USA) on a Veriti thermal cycler. This was followed by the library quality check, where the concentration of the amplified cDNA was quantified through Qubit™ DNA HS Assay Kit (Q32851, ThermoScientific, USA) in Qubit 3.0 (Q33216, ThermoScientific, USA). Agilent High Sensitivity DNA kit (5067-4626, Agilent, USA) and Agilent™ 2100 Bioanalyzer (G2939A, Agilent, USA) were employed to assess the yield and size distribution of the amplified cDNA. Sequencing was performed using ionTorrent technology. Template preparation, loading chips and sequencing, using Ion PI™ Hi-Q™ Chef Kit and Ion PI™ Chip (A43729, ThermoScientific, USA) were performed through Ion Chef and Ion Proton (4484177, ThermoScientific, USA).

Upon sequencing, we performed quality control and trimming of the FASTQ files using fastp v0.20.0. Utilizing Salmon v1.10.0, the trimmed reads were aligned to the GENCODE GRCh38.p14 transcript sequences reference Release 46, and annotation was performed based on the corresponding GENCODE GTF file. Tximport v1.30.0 was used to summarize the transcript-level estimates into gene-level expression counts. Differential analysis of gene expression was performed via DESeq2 v1.42.0. An absolute value of log2FC of 0.5 and an adjusted *p*-value of 0.05 were set. KEGG analysis was performed for all genes using DAVID, while the lncRNA enrichment analysis was achieved through ncPath platform.

## 3 Results

### 3.1 FOXO1 differential expression in HCC

**Figure 1:**
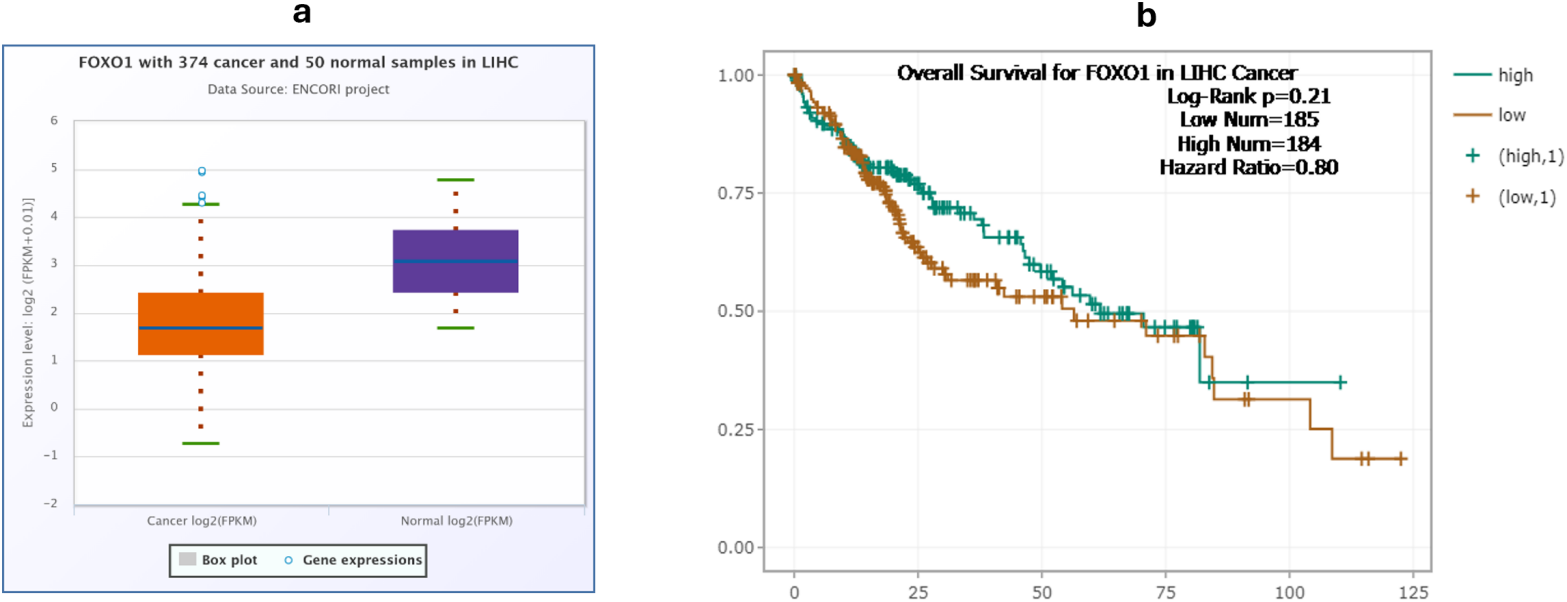
Differential expression and survival analysis of FOXO1 in HCC from ENCORI PanCancer platform (34). **(a)** Differential expression of FOXO1 in liver tissues of 374 HCC patients compared to 50 controls. **(b)** Overall survival of individuals with liver hepatocellular carcinoma (LIHC) with high versus low FOXO1 expression.

The expression data of FOXO1 in the liver tissues of 374 HCC patients and 50 control samples were gathered from ENCORI PanCancer platform. Expression analysis showed that FOXO1 is downregulated in tumor tissues of patients with a fold change of 0.47 (p-value= 9.2e-17) (**Figure 1a**). Furthermore, the overall survival rates were higher in patients with high FOXO1 expression compared to low expression with a hazard ratio of 0.8 (**Figure 1b**).

### 3.2 Identification of lncRNAs potentially regulated by FOXO1 by ChIP-Seq data analysis

ChIP-Seq data of FOXO1 performed in HepG2 cell lines were retrieved from GEO (GSE170347) to identify lncRNAs potentially regulated by FOXO1 (32). The ChIP-Seq analysis yielded 10,793 genes with FOXO1 binding sites at their promoter region within a range of ±2000 nucleotides distance from the TSS. Of those, 9,698 were identified as coding genes, while 1,095 were non-coding genes. These non-coding genes included 982 lncRNAs (**Supplementary Table 1**). Superoxide Dismutase 2 (SOD2) is a proven downstream target gene of FOXO1; thus, to confirm the rigor of our analytical procedure, as a positive control we check that SOD2 is among the list of genes with FOXO1 binding sites (34). Indeed, our ChIP-Seq data analysis revealed that SOD2 is one of the genes containing a FOXO1 binding site at the promoter region with a peak score of 105.

### 3.3 RNA-Seq analysis of lncRNAs affected by FOXO1 knockdown

**Figure 2:**
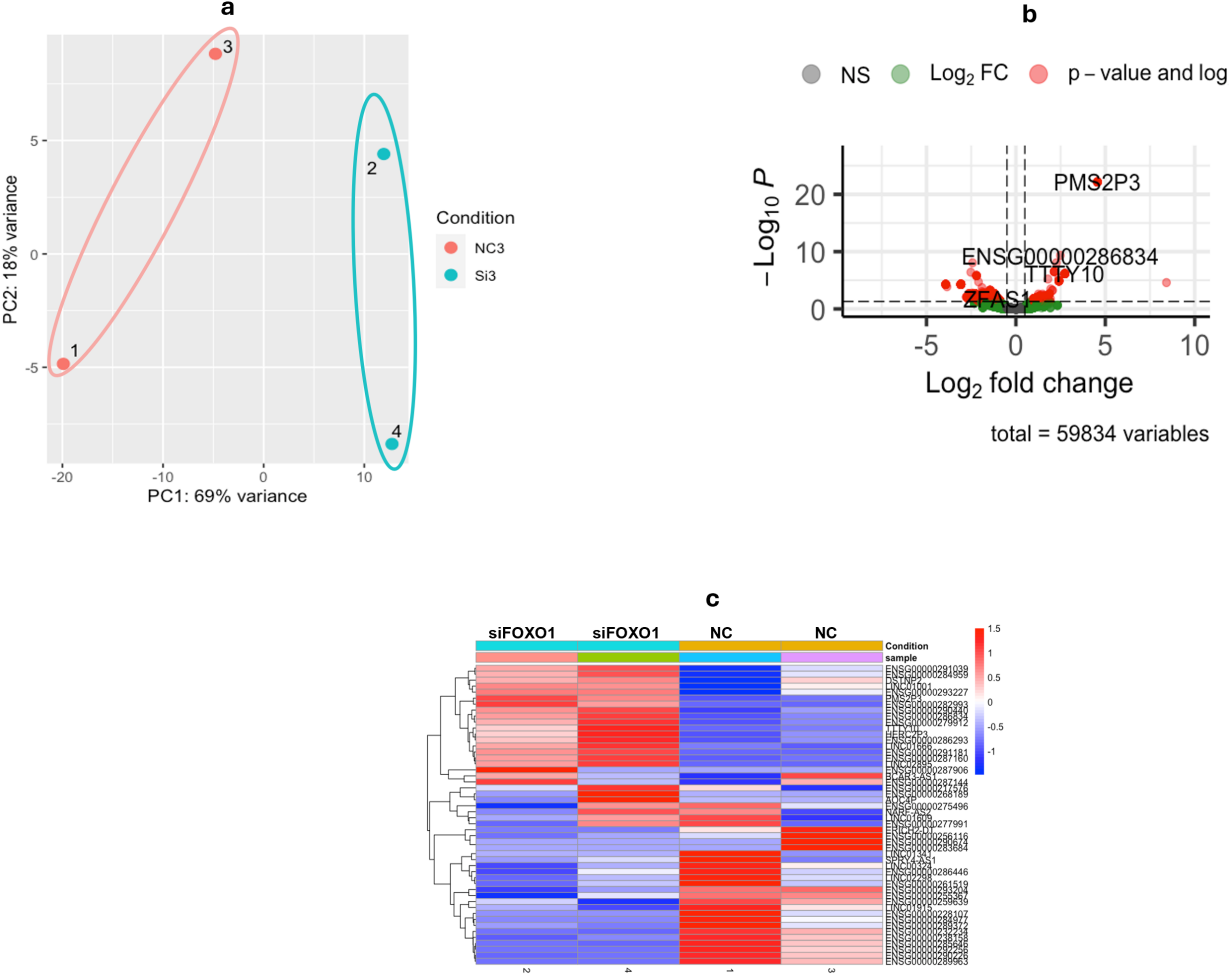
RNA sequencing data analysis. **(a)** Clustering of Samples Aligned to Manually Curated lncRNAs Reference. PCA analysis of negative control samples (NC) versus that of transfected with FOXO1 siRNA (siFOXO1) shows the clustering of NC samples on the positive side of the plot, while siFOXO1 samples clustering at the negative side. **(b)** Log(2) Fold Change of lncRNAs Expression. Volcano plot of relative expression fold change shows difference in gene expression among lncRNAs with red dots representing the upregulated lncRNAs, and green representing the downregulated lncRNAs. **(c)** Heatmap of Differentially Expressed lncRNAs Across Samples. Significantly upregulated (red) and downregulated (blue) lncRNAs are illustrated through the heatmap across NC and siFOXO1 samples.

FOXO1 was knocked down in Huh7 cells using specific siRNAs. We assessed the FOXO1 knockdown efficiency 24, 48 and 72 hours after transfection of siRNAs, and all yielded significant knockdown, nevertheless, we chose the 24 hours timepoint to minimize any secondary transcriptional changes and compensatory mechanisms. This was followed by whole transcriptome sequencing (RNA-Seq) and assessment of lncRNAs expression. The data samples clustering was examined through principal component analysis (PCA), which showed the clustering of NC samples on the left and siFOXO1 samples on the right (**Figure 2a**). Upon analyzing the samples through our pipeline, a noticeable log fold change variation was noted between the NC and the siFOXO1 samples (**Figure 2b**), with a significant differential expression (**Figure 2c**). Our analysis has shown a total of 131 differentially expressed lncRNAs of which 77 were determined to be upregulated, while 54 were downregulated (**Supplementary Table 2**).

### 3.4 Enrichment analysis of differentially expressed lncRNAs after FOXO1 knockdown

**Figure 3:**
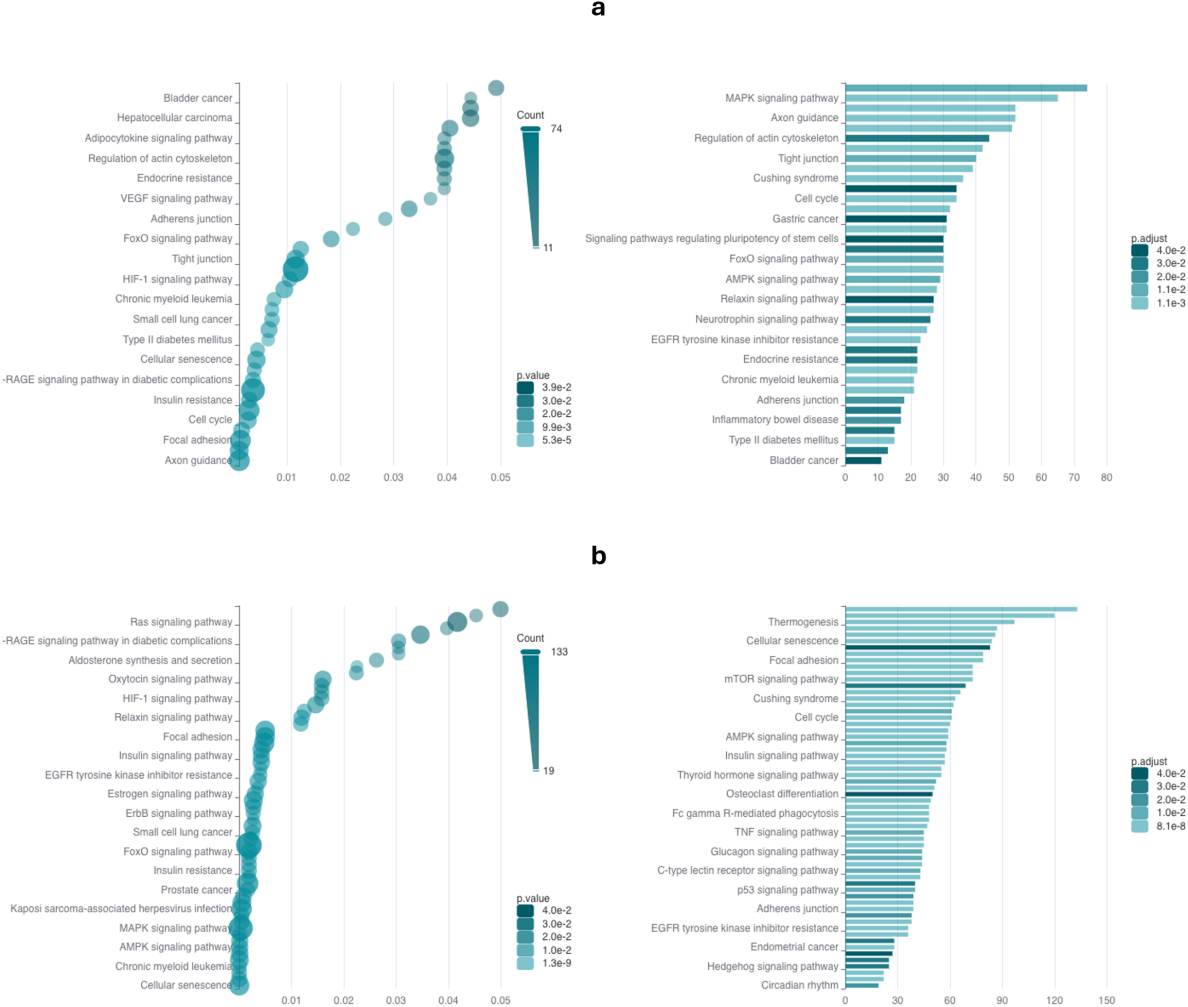
ncPath enrichment analysis of the differentially expressed lncRNAs RNA. ncPath enrichment analysis was performed for the differentially expressed **(a)** upregulated lncRNAs and **(b)** downregulated lncRNAs determined through RNA-Seq data analysis.

We performed the enrichment analysis for the list of 77 upregulated and 54 downregulated lncRNAs retrieved through our RNA-Seq analysis. The upregulated lncRNAs showed enrichment in hepatocellular carcinoma, FoxO signaling pathway, HIF1 signaling pathway, insulin resistance, EGFR tyrosine kinase inhibitor resistance, MAPK signaling pathway, cell cycle and VEGF signaling pathway (**Figure 3a**), while downregulated targets were enriched in cellular senescence, FoxO signaling pathway, EGFR tyrosine kinase inhibitor resistance, ErbB signaling pathway, Ras signaling pathway, p53 signaling, TNF signaling pathway, mTOR signaling pathway, cell cycle and glucagon signaling pathway (**Figure 3b**).

### 3.5 Identifying potential FOXO1-regulated lncRNAs based on the intersection of RNA-Seq and ChIP-Seq data

**Figure 4:**
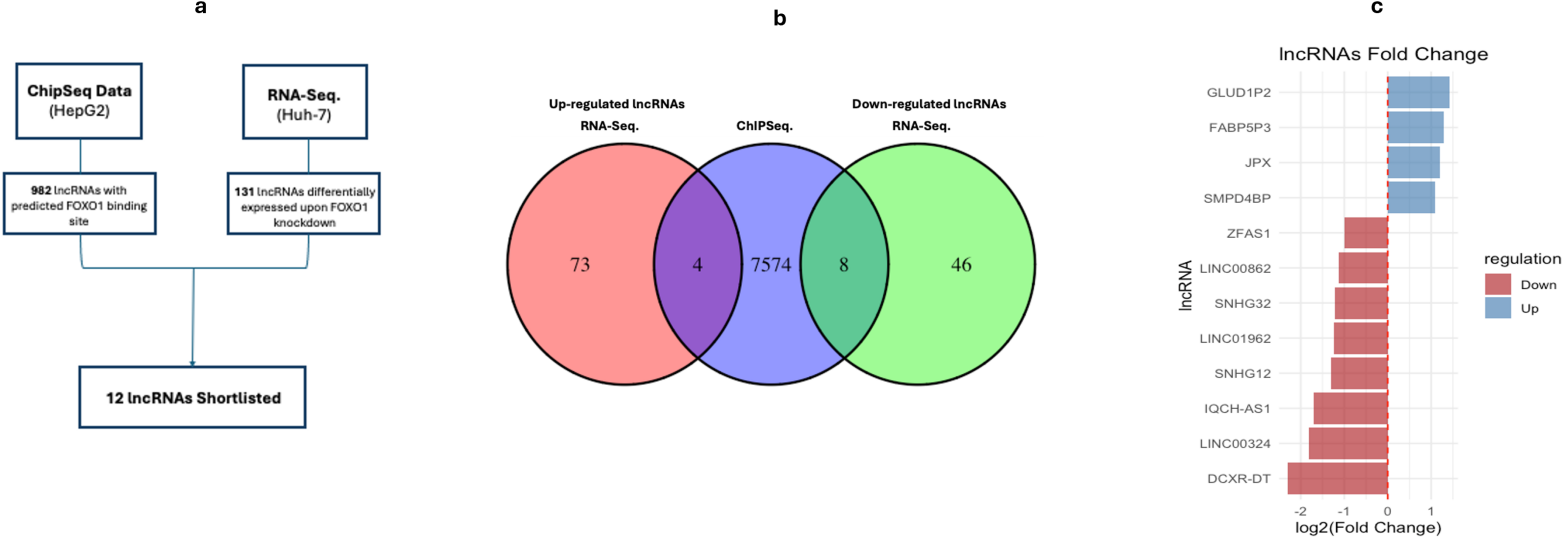
Potential FOXO1-Regulated lncRNAs Based on the Intersection of RNA-Seq and ChIP-Seq data. **(a)** Shortlisting Approach of lncRNAs Regulated by FOXO1. lncRNAs were shortlisted through examining the intersection between ChIP-Seq data of genes with FOXO1 binding sites proximal to their transcriptional start sites and our RNA-Seq data upon FOXO1 knockdown. **(b)** Venn Diagram of lncRNAs Shortlisted through ChIP-Seq and NGS Data Intersection. RNA-Seq data yielded a total of 77 significantly upregulated lncRNAs and 54 significantly downregulated lncRNAs upon FOXO1 knockdown. The common targets between RNA-Seq data and ChIP-Seq data were determined through finding the intersection, which showed 4 upregulated and 8 downregulated lncRNAs. **(c)** Fold Change of Shortlisted Four Upregulated and Eight Downregulated lncRNAs Based on RNA-Seq and ChIP-Seq Data. The bar chart shows the log2(fold change) of the shortlisted lncRNAs based on RNA-Seq and ChIP-Seq data. Four lncRNAs are upregulated with a positive log2(fold change) of at least +1, while 8 lncRNAs were down regulated with a log2(fold change) ranging between -1 to -2.

The intersection between lncRNA genes shortlisted by ChIP-Seq data and RNA-Seq data was examined (**Figure 4a**), which showed a total of 12 lncRNAs that were differentially expressed upon FOXO1 knockdown while having a FOXO1 binding site in their promoter region (**Figure 4b**). Of the 12 lncRNAs, four were upregulated (*GLUD1P2*, *FAB5P3*, *JPX*, *SMPD4BP*) showing log2(fold change) of 1.430810162, 1.294400533, 1.190768343 and 1.085042792, respectively (**Figures 4b, c**; **Supplementary Table 3**). Furthermore, eight lncRNAs were downregulated (*ZFAS1*, *LINC00862*, *SNHG32*, *LINC01962*, *SNHG12*, *1QCH-AS1*, *LINC00324*, *DCXR-DT*) with log2(fold change) of -1.002422386, -1.1273027, -1.209326334, -1.236866821, -1.305098141, -1.70573314, -1.823152463 and -2.310831734, respectively (**Figures 4b, c**; **Supplementary Table 3**). The log2(fold change) for each of the 12 shortlisted lncRNAs is illustrated in **Figure 4c**, where the upregulated lncRNAs are displayed in blue in descending order based on the log2(fold change), while the downregulated ones are in red in ascending order (**Figure 4c**).

## 4 Discussion

*FOXO1* is a key transcription factor that regulates various critical cellular functions (7, 14). One of the important functions of FOXO1 is its ability to suppress tumorigenesis through the induction of apoptosis and the inhibition of cell cycle progression (10, 35). In HCC, FOXO1 was shown to be downregulated, and patients with lower FOXO1 levels had poor survival rates (36). This highlights the importance of FOXO1 and its potential as a therapeutic target in HCC. Therefore, elucidating the downstream molecular mechanisms through which FOXO1 mediates its tumor-suppressive actions is essential for a deeper understanding of HCC pathogenesis.

In recent years, the contribution of non-coding RNAs (ncRNAs), particularly miRNAs and lncRNAs, to cancer pathogenesis and their potential as therapeutic targets has been increasingly recognized. Many studies have focused on ncRNAs that regulate FOXO1 expression and activity, demonstrating their impact on HCC progression. However, to our knowledge, the transcriptional regulation of lncRNAs by FOXO1 and their potential role in mediating FOXO1 functions in HCC has not been systematically investigated. Accordingly, in this study we focused on lncRNAs as downstream targets of FOXO1 which could potentially mediate the diverse functions of this transcription factor. Previous studies have demonstrated the value and utility of exploiting high throughput sequencing data to identify novel biomarkers in HCC and unravel potential ncRNA-gene interactions (37). To this end, we employed an integrative approach combining publicly available FOXO1 ChIP-Seq data with RNA-Seq data generated following FOXO1 knockdown to identify lncRNAs that are potentially directly regulated by FOXO1 in HCC.

Initially, we assessed the expression profile of FOXO1 by analyzing RNA-Seq data of liver tissues from 374 HCC patients and 50 control samples provided by the ENCORI PanCancer expression analysis platform, which integrates expression data from the TCGA database. Expression analysis demonstrated that FOXO1 is significantly downregulated in tumor tissues of HCC patients, which is consistent with previous studies by Wang et al. and Yang et al.; in addition, other *in vivo* studies show that FOXO1 knockdown promotes metastasis and invasion in HCC (8, 16, 38). We then compared the overall survival of HCC patients with high FOXO1 expression to those with low expression, which revealed that patients with higher FOXO1 had better overall survival (**Figure 1b**). These data agree with previously published studies that have demonstrated the correlation between FOXO1 expression and the prognosis of HCC (36, 38, 39). For example, Cui et al. found that FOXO1 was downregulated in 47% of HCC patients included in their study, with a significant inverse association between FOXO1 expression and vascular invasion (39). Furthermore, they explored the relationship between FOXO1 expression and HCC prognosis based on TCGA data extracted from 440 HCC patients revealing a positive correlation between FOXO1 expression and disease-free survival rate (39). These findings further support the tumor-suppressive role of FOXO1 in HCC and highlight its clinical relevance as a prognostic indicator.

Next, we proceeded to identify lncRNAs that are potentially regulated by FOXO1. This was achieved by performing whole transcriptome RNA-Seq after knockdown of FOXO1 in Huh-7 cells. RNA-Seq analysis revealed 131 differentially expressed lncRNAs, of which 77 were upregulated and 54 were downregulated (**Supplementary Table 2**). In parallel, to identify lncRNAs that have a FOXO1 binding site in their promoter region, we analyzed ChIP-Seq data of FOXO1 performed in the HCC cell line HepG2 provided by the ENCODE database. Our analysis of the ChIP-Seq data and shortlisting criteria revealed a list of 982 lncRNAs with a FOXO1 binding site in their promoters (**Supplementary Table 1**).

Importantly, the integration of our RNA-Seq and ChIP-Seq datasets enabled the identification of candidate lncRNAs that are not only transcriptionally altered upon FOXO1 knockdown but also potentially directly regulated by FOXO1 binding. The intersection between these two datasets yielded a final list of 12 lncRNAs. FOXO1 is known to act as both a transcriptional repressor and activator (43, 44). Therefore, we assessed the direction of regulation, revealing that eight lncRNAs were downregulated while four were upregulated (**Figure 4**). The downregulated lncRNAs are *ZFAS1, LINC00862, SNHG32, LINC01962, SNHG12, 1QCH-AS1, LINC00324*, and *DCXR-DT*, and the upregulated ones are *GLUD1P2, FAB5P3, JPX, and SMPD4BP* (**Table 1**; **Supplementary Table 3**). This bidirectional regulation suggests that FOXO1 may fine-tune oncogenic and tumor-suppressive lncRNA networks to maintain cellular homeostasis in hepatocytes. Based on our integrative analyses, we propose a working mechanistic model in which FOXO1 functions as a central transcriptional regulator of lncRNA networks in HCC. It is plausible that under physiological conditions, FOXO1 may maintain hepatocyte homeostasis by activating tumor-suppressive lncRNAs while repressing oncogenic lncRNAs. Loss or downregulation of FOXO1 in HCC may therefore lead to de-repression of oncogenic lncRNAs, such as *ZFAS1* and *SNHG12*, alongside reduced expression of lncRNAs with tumor-suppressive functions, collectively promoting proliferation, invasion, and therapeutic resistance. This proposed model provides a conceptual framework for understanding how FOXO1 dysregulation may exert broad downstream effects through lncRNA-mediated mechanisms.

**Table 1:** Summary of previous studies involving the shortlisted lncRNAs.

lncRNA	RNA-Seq	Role in liver	Other cancer types	Reference
ZFAS1	Downregulated	Oncogene	- Tumor suppressor in triple negative breast cancer - Oncogenic in nasopharyngeal carcinoma and colorectal cancer	(39–47)
JPX	Upregulated	Tumor Suppressor	- Oncogenic in lung, melanoma, esophageal squamous cell carcinoma, cervical, prostate, osteosarcoma, oral squamous cell carcinoma, colorectal cancers	(57–69)
SNHG12	Downregulated	Controversial	- Oncogenic in renal and breast cancer	(49–54)
LINC00324	Downregulated	Tumor Suppressor	- Oncogenic in several cancers	(55, 56)
LINC00862	Downregulated	NA	- Oncogenic in cervical and gastric cancers	(70)
1QCH-AS1	Downregulated	NA	- Increases chemosensitivity in thyroid cancer	(71)
GLUD1P2	Upregulated	No studies have been reported		
FAB5P3	Upregulated			
SMPD4BP	Upregulated			
DCXR-DT	Downregulated			
LINC01962	Downregulated			
SNHG32	Downregulated			

Regarding the shortlisted lncRNAs downregulated in our RNA-Seq data upon FOXO1 knockdown, *ZFAS1* has been shown to act as both an oncogene and a tumor-suppressor gene in different types of cancers, as it can achieve its role through different biological processes and pathways (**Table 1**) (40–42). *ZFAS1* knockdown caused the downregulation of MDK/ERK/JNK/P38 signaling pathway in HepG2 and SMMC7721 cell lines, and in turn decreased their malignancy; moreover, its expression was found to promote metastasis through the activation of ZEB1, MMP14, and MMP16 (23, 24). Furthermore, *ZFAS1* overexpression was correlated with donafenib and sorafenib resistance in HCC through LSD1/CoREST/p65 and PERK/ATF4 axes, respectively (44, 45). *ZFAS1* was reported to regulate different transcription factors and pathways, such as PI3K/AKT in breast cancer, LPAR1 in nasopharyngeal carcinoma, and SP1 in colorectal cancer (**Table 1**) (40, 46). Interestingly, *ZFAS1* has also been reported to act as a tumor-suppressor, where its expression was able to inhibit triple negative breast cancer through STAT3 (41). Moreover, its downregulation promoted cell migration, proliferation and invasion in breast cancer patients through PTEN/PI3K/AKT signaling pathway (47, 48). Despite its extensive characterization in HCC and other cancers, *ZFAS1* has not previously been described as a downstream transcriptional target of FOXO1, suggesting a novel FOXO1/*ZFAS1* regulatory axis that may contribute to FOXO1-mediated tumor-suppression in HCC. This highlights the necessity of experimental validation of *ZFAS1* regulation by FOXO1.

*SNHG12* has been reported to function as a competitive endogenous RNA (49, 50). Liu et al., showed that the expression of *SNHG12* in hepatic progenitor cells (WB-F344) increased their proliferation and migration through the activation of Wnt/β-catenin pathway (**Table 1**) (51). In HCC, Lan et al. found *SNHG12* to promote tumorigenesis and metastasis through sponging miR-199, thereby regulating MLK3 expression and the NF-κB pathway activity (52). Conversely, *SNHG12* was shown to inhibit cell proliferation and metastasis in intrahepatic cholangiocarcinoma through the miR-199a-5p/Klotho axis (53), and was reported as an oncogene in both renal and breast cancers (54, 55). To our knowledge, no study has described the upstream regulators of *SNHG12* nor specifically linked its expression to the transcription factor FOXO1. Our findings therefore identify FOXO1 as a potential upstream regulator of *SNHG12*, pointing to possible new insights into the transcriptional control of *SNHG12*-mediated oncogenic signaling in liver cancer.

*LINC00324* has been reported as both a tumor-suppressor and an oncogene (49). A study by Zhang et al. has shown that *LINC00324* enables p53 transactivation, as both genes are located on the same chromosome, leading to destabilization of the p53–SET complex. A homodeletion was found to cause a downregulation in *LINC00324* expression within 2.44% of patients with HCC (56). On the other hand, another study by Gao et al. illustrated that *LINC00324* was highly expressed in HCC patients, and reported that FasL was promoted upon *LINC00324* expression through PU box binding protein, enabling cancer progression (54, 57). Furthermore, in several cancers low levels of *LINC00324* were positively correlated with aggressiveness of clinical presentation and worsening of overall survival (56). These conflicting observations highlight the context-dependent functions of *LINC00324* and suggest that FOXO1-mediated regulation may influence its role in HCC.

*JPX*, also known as *XIST* activator, exists on the X-chromosome in its X-inactivation center (49). It was also found to be of lower expression in HCC specimens, with its overexpression resulting in a decrease in HepG2 cell growth achieved through SOX6 (**Table 1**) (58, 59). In contrast to its reported tumor-suppressive role in HCC, *JPX* has been reported to facilitate tumorigenesis, migration, and metastasis in many types of cancer, such as lung cancer, melanoma, esophageal squamous cell carcinoma, cervical cancer, prostate cancer, osteosarcoma, oral squamous cell carcinoma, and colorectal cancer through various pathways (**Table 1**) (60–70). The identification of *JPX* as a FOXO1-regulated lncRNA suggests that FOXO1 may modulate X-linked lncRNA networks involved in hepatocarcinogenesis.

*LINC00862* has been suggested to act as an oncogenic lncRNA that can be used as a prognostic and diagnostic biomarker in cervical and gastric cancers through a bioinformatic pan-cancer study (71). It was also reported as a potential predictive biomarker for immunotherapeutic efficacy (71). *1QCH-AS1* has only been studied in thyroid cancer, where its expression increased the chemosensitivity of the cancer cells to doxorubicin (**Table 1**) (49, 72). Nevertheless, there is no literature available to classify its role in the liver or other organs (**Table 1**).

Concerning the shortlisted lncRNAs upregulated in our data after FOXO1 knockdown, *GLUD1P2*, known as glutamate dehydrogenase 1 pseudogene 2, has been reported to have high expression levels in the liver (49, 73). *DCXR-DT*, on the other hand, is a divergent lncRNA which has no previously identified role as it is a new class of lncRNAs yet to be explored (74). Similarly, *FAB5P3*, *LINC01962, SNHG32* and *SMPD4BP* are novel lncRNA targets with no literature reporting their function. Thus, the identification of these poorly characterized lncRNAs as FOXO1-regulated targets represents a significant contribution of this study and underscores the need for their functional and mechanistic characterization.

Our enrichment analysis has shown that the upregulated lncRNAs from our RNA-Seq data (**Supplementary Table 2**) are involved in various pathways and biological contexts such as in the development of HCC, FoxO signaling pathway, HIF1 signaling pathway, insulin resistance, EGFR tyrosine kinase inhibitor resistance, MAPK signaling pathway, cell cycle and VEGF signaling pathway (**Figure 3a**). On the other hand, our list of downregulated lncRNAs were enriched in cellular senescence, FoxO signaling pathway, EGFR tyrosine kinase inhibitor resistance, ErbB signaling pathway, Ras signaling pathway, p53 signaling, TNF signaling pathway, mTOR signaling pathway, cell cycle and glucagon signaling pathway (**Figure 3b**). This highlights the possible relatedness of our shortlisted lncRNAs to integral pathways and biological processes that contribute to the development of HCC, whether directly or indirectly. The importance of FOXO1 in the regulation of those lncRNAs is also underlined by the fact that both up- and downregulated lncRNAs showed significant enrichment in the FoxO signaling pathway (**Figure 3**). Moreover, the enrichment of both up- and downregulated lncRNAs within the FoxO signaling pathway suggests the existence of feedback regulatory loops that may modulate FOXO1 activity in HCC. Taking all our findings together, we suggest that these lncRNAs are potential downstream targets of FOXO1 and are promising candidates for further studies that would aim to comprehend their role in mediating FOXO1 functions.

Despite the strengths of the integrative RNA-Seq and ChIP-Seq approach, this study has several limitations. First, direct transcriptional regulation and functional relevance of individual lncRNAs will require experimental validation. Techniques such as qRT-digital PCR, FOXO1 overexpression/knockdown, luciferase reporter assays, and ChIP-PCR, are necessary to confirm and validate the direct interaction between FOXO1 and our shortlisted lncRNAs. The use of short-read sequencing may limit accurate lncRNA isoform annotation, and future studies incorporating long-read sequencing technologies such as Oxford Nanopore may provide greater resolution. Second, the use of a single HCC cell line limits the generalizability of the findings. Therefore, in our future work, we will aim to validate the direct regulation of our 12 candidate lncRNAs by FOXO1 in other liver cancer models. Third, given the molecular heterogeneity of HCC, FOXO1-lncRNA regulatory interactions may vary across different cellular contexts, tumor stages, and etiological backgrounds, including viral versus non-viral HCC. Valuable insights can be gained from examining tissue and serum samples collected from patients with HCC to explore the signature of these lncRNAs within the heterogeneous environment of liver cancer. Interesting correlations may be drawn between the signature of these lncRNAs and the patients’ clinical data such as staging and survival rates. This will enable us to ensure the diagnostic and prognostic potential of our shortlisted lncRNAs and further validate the biological and translational significance of FOXO1-mediated lncRNA regulation in HCC (75, 76).

Future research should be directed towards examining the potential applications of lncRNAs in translational medicine. Since RNA-based therapy and mRNA vaccines are currently a rapidly expanding field in hepatotropic diseases, it would also be worth exploring the use of lncRNAs in vaccination against HCC. While lncRNAs represent attractive therapeutic and diagnostic targets, their clinical translation faces challenges related to tissue specificity, delivery, and stability. Therefore, careful validation in clinically relevant models will be essential before FOXO1-regulated lncRNAs can be considered for therapeutic applications.

In conclusion, through our integrated study of ChIP-Seq and whole transcriptome RNA-Seq data, we have shortlisted 12 lncRNAs, namely, *GLUD1P2, FAB5P3, JPX, SMPD4BP, ZFAS1, LINC00862, SNHG32, LINC01962, SNHG12, 1QCH-AS1, LINC00324*, and *DCXR-DT*, that are potentially regulated by the tumor-suppressor transcription factor, FOXO1, in HCC. Our findings necessitate the initiation of research that would explore the role of these lncRNAs and how they affect the progression of certain diseases, such as HCC. These lncRNAs represent promising candidates for further validation through *in vitro* and *in vivo* studies with a potential application as prognostic markers for liver diseases as well as novel therapeutic targets. Collectively, our findings expand the current understanding of FOXO1-dependent transcriptional networks by identifying lncRNAs as key downstream mediators of FOXO1 tumor-suppressive function in HCC.

## Data Availability

The original contributions presented in the study are publicly available. This data can be found here: European Nucleotide Archive (ENA), PRJEB107983, https://www.ebi.ac.uk/ena/browser/view/PRJEB107658.
