# Supplementary figures and images for: Pilot study: predicting the interplay between FOXO1 and its downstream long non-coding RNAs in HCC
Source: Front Oncol. 2026 Feb 16;16:1692980. doi: 10.3389/fonc.2026.1692980 (PMC12950588; doi:10.3389/fonc.2026.1692980)

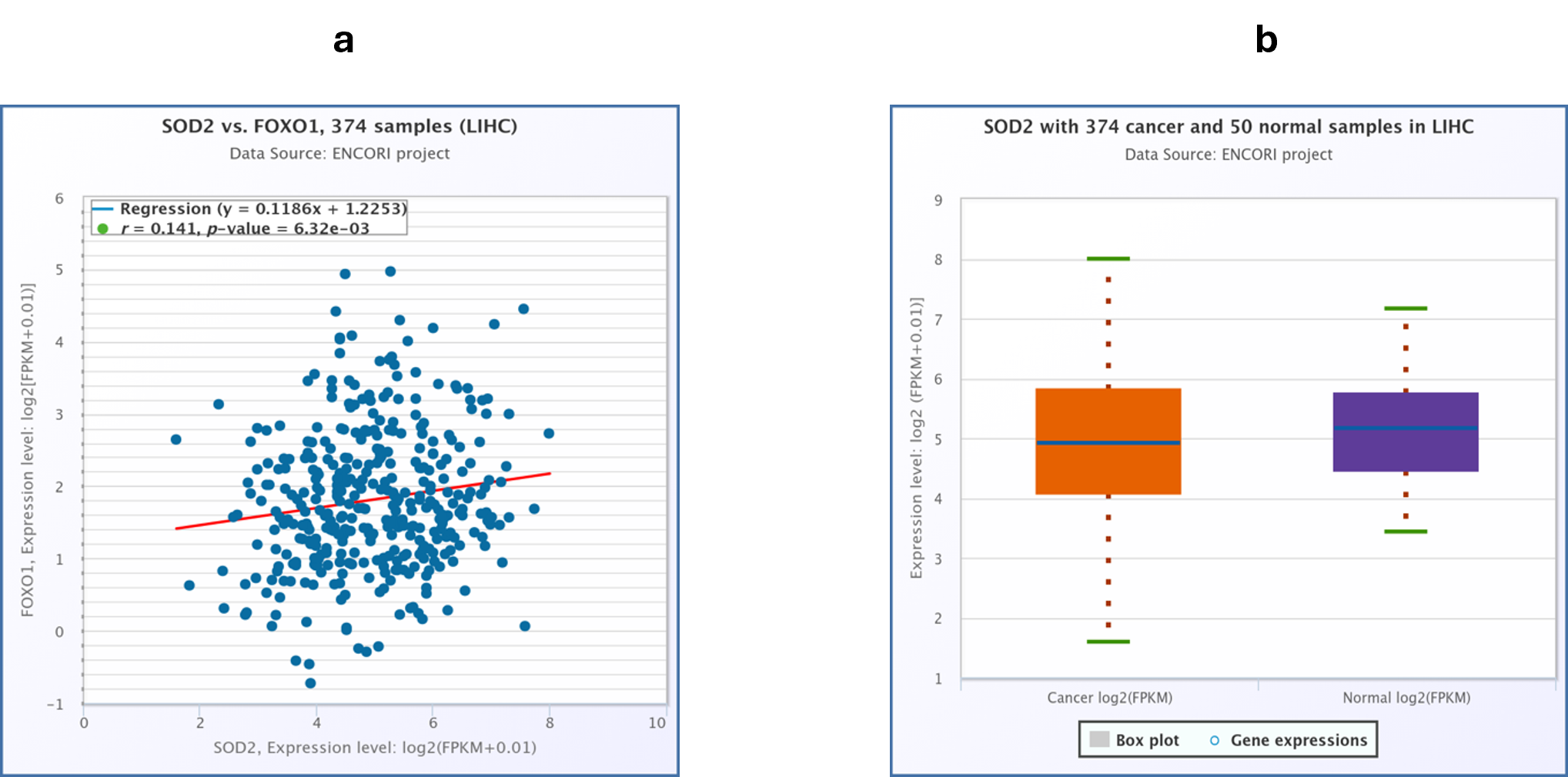

Supplement: Supplementary file 2 [file Image1.tif]
